# Electron-Transfer Properties of Phenyleneethynylene Linkers Bound to Gold via a Self-Assembled Monolayer of Molecular Tripod

**DOI:** 10.3390/molecules23112893

**Published:** 2018-11-06

**Authors:** Toshikazu Kitagawa, Takashi Kawano, Takahiro Hase, Ikuma Hayakawa, Katsuyuki Hirai, Takao Okazaki

**Affiliations:** 1 Department of Chemistry for Materials, Graduate School of Engineering, Mie University, Tsu, Mie 514-8507, Japan; doragonnaito0723@yahoo.co.jp (T.K.); hase@kuroganekasei.co.jp (T.H.); alc2014jp@gmail.com (I.H.); okazaki@chem.mie-u.ac.jp (T.O.); 2 Organization for the Promotion of Regional Innovation, Mie University, Tsu, Mie 514-8507, Japan; hirai@chem.mie-u.ac.jp

**Keywords:** molecular tripod, self-assembled monolayer, electron transfer, adamantane, ferrocene

## Abstract

The three-point adsorption of tripod-shaped molecules enables the formation of robust self-assembled monolayers (SAMs) on solid surfaces, where the component molecules are fixed in a strictly upright orientation. In the present study, SAMs of a rigid molecular tripod consisting of an adamantane core and three CH_2_SH groups were employed to arrange ferrocene on a gold surface through oligo(*p*-phenyleneethynylene) linkers. Cyclic voltammetry of the monolayers demonstrated high surface coverage of ferrocene, yet the molecular interaction among adjacent ferrocene units was negligible. This was because of the extended intermolecular distance caused by the bulky tripod framework. The rates of electron transfer from the ferrocene to the gold surface through different linker lengths were determined by electrochemical measurements, from which the decay factor for oligo(*p*-phenyleneethynylene) wire was evaluated.

## 1. Introduction

Self-assembled monolayer (SAM) of thiolates on a gold surface, produced by the tight Au–S bonding, creates a secure molecule–metal junction [1,2,3,4]. The thermodynamically favored chemisorption of molecules enables monolayer formation simply by forcing thiols to make contact with a clean gold surface. The resultant dense SAMs have been applied to sensors [5,6], molecular machines [7,8], and molecular electronic devices [9,10,11]. In particular, a SAM of electron-transporting molecules could serve as an excellent junction between the device molecule and a metal electrode.

The component molecules in a densely packed SAM, however, are subjected to severe steric and electrostatic interactions, which can exert an undesirable mutual intermolecular influence that can potentially alter the structural and electronic features of the original molecules in their isolated state. This would hamper the design and utilization of the single-molecule functionalities of the monolayers.

**Figure 1:**
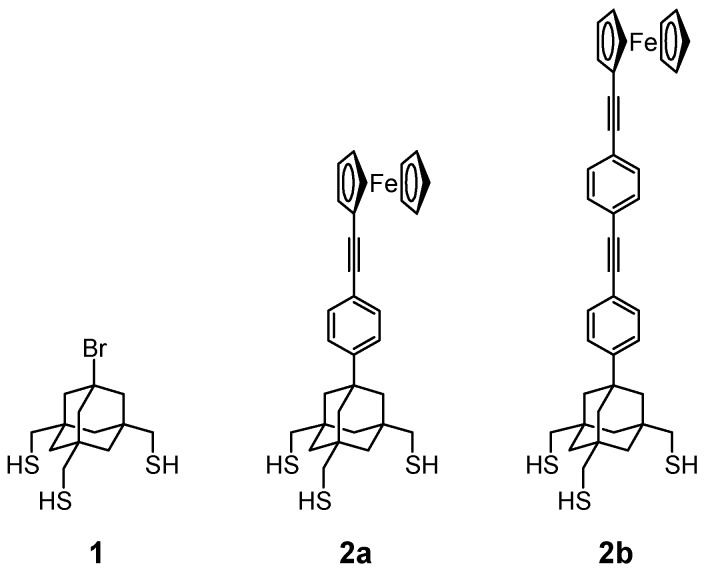
Tripod-shaped trithiols with an adamantane core.

The use of tripod-shaped anchors may be, due to their surface-demanding nature, effective in avoiding such interactions by isolating the molecules within a SAM. A variety of SAMs composed of tripodal trithiols have been reported using the sp^3^-hybridized carbon [10,12,13] or silicon [14] atom as a central tetrahedral core. Other tripods have utilized the rigid carbon framework of adamantane, in which the four bridgehead bonds extend in the tetrahedral direction [15,16,17,18,19,20,21]. A cyclohexane ring, which has a partial structure of adamantane, was also used as a core carbon framework for three thiol legs [22,23]. Previously, we reported a tripod molecule **1** [16,17,18,19,21] and its ferrocenyl derivative **2a** [18,19,20], consisting of a rigid adamantane core and three CH_2_SH legs (Figure 1). X-ray photoelectron spectroscopy (XPS) studies supported the three-point adsorption of these molecules on the Au(111) surface [20], and a perpendicular orientation of the molecule was shown by DFT optimization [21].

**Figure 2:**
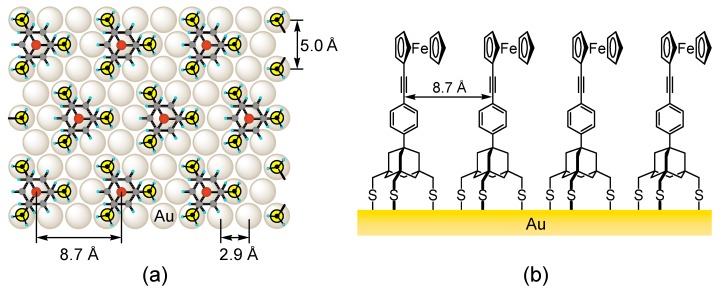
(**a**) Proposed molecular orientation of the SAMs of **1** on the Au(111) surface; and (**b**) the schematic diagram of the SAM of ferrocene-terminated trithiol **2a** on a gold surface.

Based on the results of scanning tunneling microscopy (STM) studies, the SAM of **1** is hexagonally arranged with a closest molecular distance of 8.7 Å (Figure 2a) [16]. This distance is significantly larger than that normally observed for the SAMs of linear alkanethiols (5.0 Å) [2,4,24] and allows molecule **2a** to arrange in the same density, wherein neighboring ferrocenyl groups are isolated from each other (Figure 2b) [20]. This feature is highly desirable for the study of the electron transport properties of π-conjugated molecular wires, such as oligo(*p*-phenyleneethynylene) or –(C_6_H_4_-C≡C)*_n_*–. The connection of the straight, rod-like structure of this wire to the adamantane tripod is of particular interest, since the wire may be kept upright in the SAM without intermolecular contact.

In this paper, we report the electron transfer behavior of *p*-phenyleneethynylene bridges joining ferrocene and gold substrate using the SAMs of **2a** and **2b** via electrochemical techniques. Reliable single-molecule properties were expected, owing to the independence of each component molecule from neighboring molecules. The decay of the electron transfer rate through the molecule was discussed by comparing the results from the two SAMs.

## 2. Results and Discussion

### 2.1. Reductive Desorption

The SAMs were formed by immersing a Au(111) substrate, prepared by vacuum deposition of gold on mica, into a dichloromethane solution of **2a** or **2b** at ambient temperature. Thiol–gold SAMs generally undergo desorption of thiolate ion by electrochemical reduction according to Equation (1) [25,26,27,28,29,30,31].

RS–Au(s) + e^−^ → RS^−^ + Au(s) (1)

where Au(s) represents the gold atom on the solid surface.

**Figure 3:**
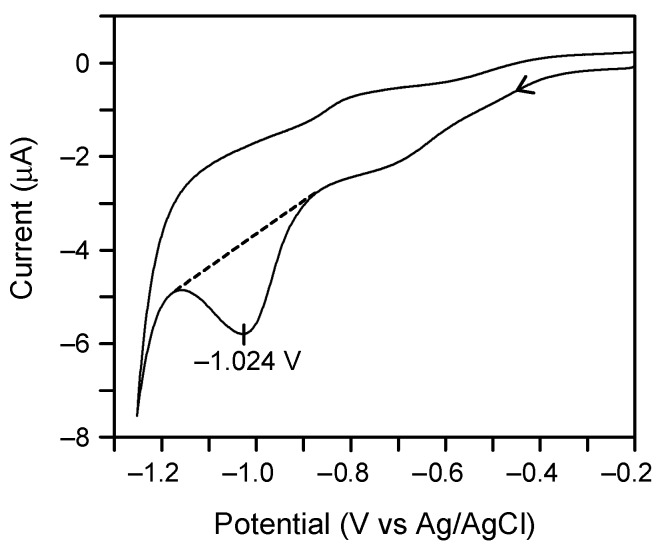
Reductive desorption of the SAM of **2b** on Au(111), as observed by cyclic voltammetry using the surface-modified gold substrate as a working electrode in 0.5 M aqueous KOH. The scan rate was 0.02 V/s. The geometric area of the working electrode was 0.152 cm^2^. The charge for reductive desorption was calculated from the area below the dotted line.

Previously, we reported that the SAMs of **1** and **2a** show a reductive desorption peak in cyclic voltammetry [16,20]. The cyclic voltammetry of the SAM of **2b** at negative potentials in aqueous KOH also showed an irreversible reduction peak at −1.024 V versus Ag/AgCl (Figure 3). The peak potential (*E*_p_), charge density of the reductive wave (*Q*_red_), and full width at half-maximum (Δ*E*_fwhm_) of related SAMs are summarized in Table 1.

The SAMs of long-chain *n*-alkanethiols, such as *n*-dodecanethiol, are known to show a large negative *E*_p_ (<−1.0 V) because the adsorbed molecules resist desorption due to strong attractive interactions between closely neighbored, van der Waals-contacting alkyl groups [30]. Although such interactions are small in the SAMs of tripod molecules **2a** and **2b**, large negative reduction potentials were similarly observed for these molecules. This can be attributed to tighter binding of the molecules to the substrate by the three S–Au bonds.

The small Δ*E*_fwhm_ for *n*-dodecanethiol can also be ascribed to the strong attractive interaction between neighboring alkyl chains [30], while the much larger values observed for tripod trithiols indicate the insignificance of such interactions. The *Q*_red_ of the SAM of **2b** is comparable to that of **2a**, indicating that, despite the extended molecular length of **2b**, its SAM is as densely packed as in the case of **2a**.

### 2.2. Oxidation of the Ferrocenyl Group

The cyclic voltammograms of the SAMs of **2a** and **2b** at positive potentials showed reversible redox waves at approximately 0.4 V versus Ag/AgNO_3_, owing to the single-electron oxidation of the ferrocenyl group. The redox charges, calculated from the mean value of oxidation and reduction peak areas, were *Q*_ox_ = 24 ± 2 μC/cm^2^ for both SAMs. This corresponds to a surface coverage (*Γ* = *Q*_ox_/*F*, where *F* is the Faraday constant) of 2.5 × 10^–10^ mol/cm^2^ and indicates that the molecules were packed as densely as in the SAM of **1**. If each of the **2a** and **2b** molecules are adsorbed by three sulfur atoms, the reductive desorption charge is expected to be 3*FΓ* = 72 μC/cm^2^. Considering that approximately 30% of the additional reductive charge is often observed upon desorption of S–Au SAMs due to the change in double layer capacitance, a *Q*_red_ value of 94 μC/cm^2^ is expected. The observed values for *Q*_red_ for the SAM of **2a** and **2b** approximate this value, which supports a secure three-point adsorption.

At sufficiently low scan rates (<0.1 V/s), the full width at half-maximum (Δ*E*_fwhm_) and the cathodic to anodic peak separation (Δ*E*_pp_) approximated those predicted for an ideal Nernstian redox system (Δ*E*_fwhm_ = 90.6 mV and Δ*E*_pp_ = 0 mV) [32], which indicated that each ferrocenyl group in these SAMs was well isolated from its neighboring molecules. In this scan rate region, Δ*E*_pp_ was not affected by the scan rate variation.

**Figure 4:**
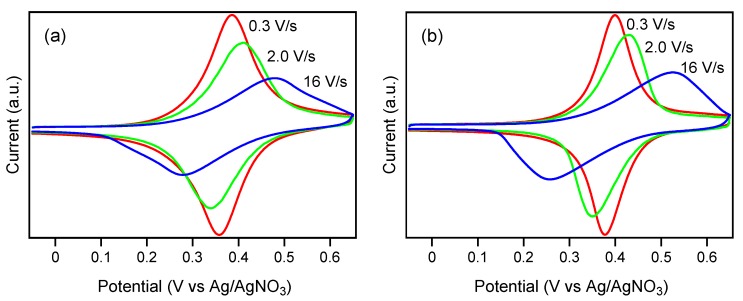
Cyclic voltammograms of the SAM of (**a**) **2a** and (**b**) **2b** recorded at different scan rates in CH_2_Cl_2_ containing 0.1 M TBAP as a supporting electrolyte. The geometric area of the working electrode was 0.152 cm^2^.

**Figure 5:**
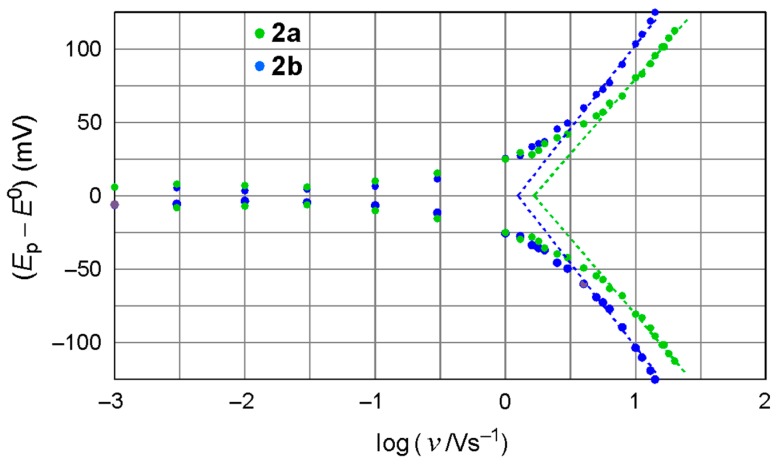
Plot of *E*_p_ − *E*^0^ versus the logarithm of scan rate for the SAMs of **2a** and **2b** on Au(111). *E*^0^ was 0.371 and 0.392 V versus Ag/Ag^+^ for **2a** and **2b**, respectively.

Upon increasing the scan rate over 0.1 V/s, both SAMs showed a gradual increase in Δ*E*_pp_ (Figure 4), which is typical of a kinetic outcome involving the rate of electron transfer through adsorbed material. Figure 5 illustrates the plots of the anodic and cathodic peak potentials relative to the formal potential *E*^0^ on the natural logarithm of the scan rate. Based on the increases in Δ*E*_p_ as a function of the scan rate, the rate constant of electron transfer (*k*_ET_) was estimated using Laviron’s Equation (2) [33], resulting in values of 40 ± 5 and 28 ± 4 s^–1^ for the SAMs of **2a** and **2b**, respectively.

*k*_ET_ = *αnFv*_0_/*RT* (2)

where *n* is the number of electrons transferred per molecule (*n* = 1 in the present case), *F* is the Faraday constant, *R* is the gas constant, and *T* is the temperature (298 K). *v*_0_ is the scan rate at the intercept of the fit line (dashed lines in Figure 5) with the horizontal line *E*_p_ − *E*^0^ = 0. The transfer coefficient, *α*, was set to 0.5.

### 2.3. Dependence of the Electron Transfer Rate on Distance

In general, the electron transfer rate decays exponentially with distance, as follows.

**Figure 6:**
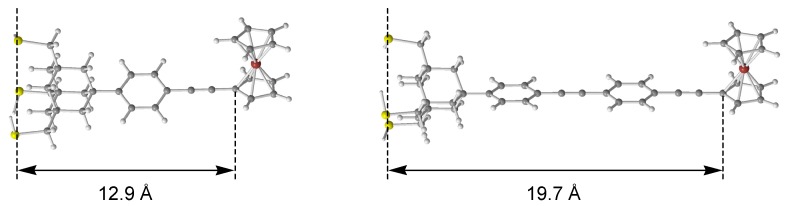
Structures of trithiol **2a** (**left**) [20] and **2b** (**right**) optimized at the B3LYP/3-21G(d)-LANL2DZ level.

*k*_ET_*= k*_0_ exp(−*βr*) (3)

where *k*_0_ is the preexponential factor and *β* is the decay constant. The distances (*r*), taken from the plane determined by the three sulfur atoms to the carbon atom in the ferrocene attached to the ethynyl carbon, were estimated to be 12.9 and 19.7 Å in the SAMs of **2a** and **2b**, respectively, from the DFT optimized structure of the trithiols (Figure 6). On the basis of the deceleration of the electron transfer from 40 s^−1^ (**2a**) to 28 s^−1^ (**2b**), the decay constant (*β*) was derived to be 0.05 Å^−1^.

**Figure 7:**
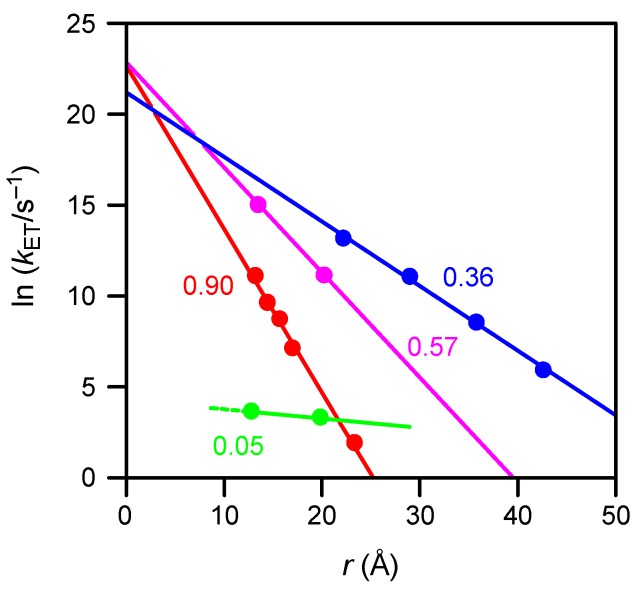
Plots of ln *k*_ET_ versus distance for SAMs of molecular wires bridging ferrocene and gold via S–Au bonding. Green (this work), blue [34], and purple [35]: oligo(*p*-phenyleneethynylene) wire. Red [36]: linear alkylamide wire. Values of *β* are indicated.

The *β* values for similar, but adamantane-free, oligo(*p*-phenyleneethynylene) wires substituted with methyl and propoxy groups were reported by Creager et al. (*β* = 0.36 Å^−1^, *n* = 3–6) [34] and by Sachs et al. (*β* = 0.57 Å^−1^, *n* = 2, 3) [35] using non-electroactive diluent thiols. The *r*–ln *k*_ET_ plot in Figure 7 shows their data, together with our results and those reported by Creager for linear alkyl chain wires, FcCONH(CH_2_)*_m_*S–Au(s) (*m* = 7–10 and 15) [36].

A significant feature of our data is the extremely slow electron transfer, by a factor of approximately e^–10^ (<10^–4^), compared with that reported for adamantane-free oligo(*p*-phenyleneethynylene) wires. The reason could be the insertion of an aliphatic adamantyl group into our molecule. Another important point is that although all lines in Figure 7, except for that of the present work, converge to ln *k*_ET_ = 23 at *r* = 0, which corresponds to the common limit at zero distance, our data extrapolates to a much lower value, ln *k*_ET_~4. This could also be caused by the presence of the adamantane tripod acting as a resistor between the end of the conjugated wire and the gold.

The cause of the very small *β* (0.05 Å^−1^) of our linker is unclear. A possible cause of the large difference between this value and other oligo(*p*-phenyleneethynylene) wires in Figure 7 could be the electrolyte used for our measurement (TBAP in CH_2_Cl_2_), which has a lower polarity than either aqueous HClO_4_ or NaClO_4_ that was used in other studies [34,35].

## 3. Materials and Methods

### 3.1. Materials

For electrochemical experiments, water was purified using a Millipore Simplicity 185 Water System (18 MΩ cm resistivity). Other solvents for electrolyte preparation and the rinsing of glassware were of the highest purity commercially available and were used without further purification. Gold (99.99%) was obtained as a 0.8-mm wire. Mica was purchased from Nilaco (Tokyo, Japan) in the form of a 0.40–0.45-mm natural sheet.

**Scheme 1:**
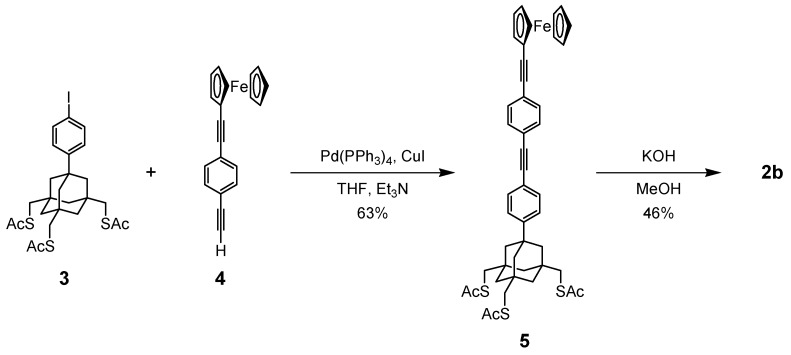
Synthetic route to elongated trithiol **2b**.

Trithiol **2a** was prepared by the method we reported earlier [20]. Compound **2b** was synthesized by Sonogashira coupling reaction of iodopheny-terminated trithioacetate **3** [16,20] and ferrocene derivative **4** [37] to give **5**, followed by the methanolysis of the three AcS groups (Scheme 1). Details of the synthetic procedure are provided in the Supplementary Materials.

### 3.2. Preparation of Self-Assembled Monolayers on Gold

Gold substrates with a (111) surface were prepared via vacuum vapor deposition of gold (99.99%) onto freshly cleaved mica sheets (0.05 mm thickness, 6 × 6 cm) under high vacuum (<10^−3^ Pa) at a substrate temperature of 580 °C. The deposition was performed at an evaporation rate of 1.0–1.5 nm/s until a gold thickness of 200 nm was reached. The obtained substrate was cut into 1 × 2 cm pieces and annealed at 530 °C in a furnace for 8 h under air to remove the surface contamination and to minimize defects. SAMs were formed by soaking the substrate in a 0.1 mM solution of trithiols in CH_2_Cl_2_ for at least 24 h.

### 3.3. Electrochemical Measurements

Glassware (cells and pipettes, etc.) used for electrochemical measurements were soaked in 10% potassium hydroxide in 2-propanol for 24 h to remove surface organic contaminants, washed thoroughly with deionized water, and dried under air at 100 °C before use. The surface-modified gold substrate was mounted at the bottom of a cone-shaped cell using an O-ring to serve as a working electrode. The area of the electrode exposed to the electrolyte was 0.152 cm^2^ (i.e., a 4.4 mm diameter circle). Reductive desorption was recorded with aqueous 0.5 M KOH using an Ag/AgCl/sat. KCl reference electrode (Supplementary Materials, Figure S5a). Redox waves of a ferrocenyl group were observed using a CH_2_Cl_2_ solution containing 0.1 M tetrabutylammonium perchlorate (TBAP) and an Ag/AgNO_3_ (0.01 M in CH_3_CN) reference electrode (Supplementary Materials Figure S5b). The electrolyte solution in the cell was de-aerated by bubbling argon for 10 min before scanning. Voltammograms were recorded using a ALS600C electrochemical analyzer (BAS, Tokyo, Japan).

### 3.4. Theoretical Calculations

Results of DFT calculations [38] for **2a** were reported earlier [20]. Calculations for **2b** were performed in a similar manner using the Gaussian 03 program [39]. Geometry optimization was carried out using the B3LYP method and general basis set (Gen keyword). The C, H, and S atoms were calculated with the 3-21G(d) basis set, whereas Fe was calculated with the LANL2DZ (5D, 7F) basis set. The results of optimization are shown in Supplementary Materials, Table S1. The obtained geometry was verified by frequency calculations to have no imaginary frequencies.

## 4. Conclusions

Tripod-shaped trithiols, **2a** and **2b**, bearing a ferrocenyl group via mono and bis(*p*-phenyleneethynylene) linker formed SAMs on Au(111) surface that featured negligible interactions among neighboring ferrocenyl groups, regardless of the high coverage. These SAMs showed *k*_ET_s [40 ± 5 s^–1^ (**2a**) and 28 ± 4 s^–1^ (**2b**)] that are more than four orders of magnitude smaller than the values reported for linkers of the same type and length. A very small decay constant, *β* = 0.05 Å^−1^, was also observed. These findings can be explained by the fact that a saturated framework of adamantane insulates conjugated *p*-phenyleneethynylene wire and gold. Another possible reason is the low polarity of the electrolyte we employed, compared with those used in other works. Studies on the electron transfer properties of further elongated oligo(*p*-phenyleneethynylene) linkers are in progress. Also, it would be valuable to elucidate the nature of the “resistance” posed by the adamantane framework by the use of directly linked ferrocene–adamantane tripod.

## Figures and Tables

**Table 1:** Peak potential (*E*_p_), charge density (*Q*_red_), and full width at half-maximum (Δ*E*_fwhm_) for the electrochemical reductive desorption of SAMs derived from trithiols and dodecanethiol on Au(111). ^a^

Thiol	*E*_p_	*Q*_red_^b,c^	Δ*E*_fwhm_^c^	Reference
	(V vs. Ag/AgCl)	(μC/cm^2^)	(mV)	
**1**	−1.088	100	91	[16]
**2a**	−0.977	91	125	[20]
**2b**	−1.024	93	134	This work
*n*-C_12_H_25_SH	−1.084	100	20	[16]

^a^ Measured by cyclic voltammetry in 0.5 M aqueous KOH. The scan rate was 0.02 V/s. All measurements were performed using the same instrumentation and protocol in the same laboratory to allow comparison. ^b^ Calculated from the reduction peak area. ^c^ The uncertainty due to sample-to-sample variations was ±10%.

## Supplementary Materials

Supplementary materials including synthetic procedures, figures of NMR spectra and cyclic voltammetry cells, and the results of DFT calculations are available online.
