# Supplementary material for: Electron-Transfer Properties of Phenyleneethynylene Linkers Bound to Gold via a Self-Assembled Monolayer of Molecular Tripod
Source: Molecules. 2018 Nov 6;23(11):2893. doi: 10.3390/molecules23112893 (PMC6278298; doi:10.3390/molecules23112893)
Supplement: Supplementary file 1 [file molecules-23-02893-s001.pdf]

Supporting Information

Electron-Transfer Properties of Phenyleneethynylene Linkers Bound to Gold via a Self-Assembled Monolayer of Molecular Tripod

Toshikazu Kitagawa, Takashi Kawano, Takahiro Hase, Ikuma Hayakawa, Katsuyuki Hirai and Takao Okazaki

Table of Contents page

Synthetic Procedures and Characterization Data S2

Figure S1. ^{1}H NMR spectrum of **5** S4

Figure S2. ^{13}C NMR spectrum of **5** S4

Figure S3. ^{1}H NMR spectrum of **2b** S5

Figure S4. ^{13}C NMR spectrum of **2b** S5

Figure S5. Cyclic voltammetry cells S6

Table S1. Cartesian coordinates and energies for the DFT-optimized structure of **2b**. S7

Synthetic Procedures and Characterization Data

Chemicals and equipment. Anhydrous solvents used for synthesis were prepared using standard methods. Other reagents for synthesis were used as received. ^{1}H NMR spectra were obtained using a JEOL JNM-A500 (500 MHz) or a JNM-AL300 (300 MHz) instrument. Compound **3** was prepared via a previously reported method [1,2]. Compound **4** was synthesized according to the method of Chidsey [3]. ^{13}C NMR spectra were measured using either a JEOL JNM-A500 (126 MHz) or a JNM-AL300 (75 MHz) instrument. The IR spectra were recorded on a JASCO FT/IR-4200 spectrophotometer. High-resolution mass spectrometry was performed using an Applied Biosystems Voyager-DE PRO or an AB SCIEX TOF/TOF 4800 spectrometer. Preparative gel permeation chromatography was performed in a recycle mode using a Shodex H-2001 column (20 mm × 50 cm).

1-[4-[4-(Ferrocenylethynyl)phenylethynyl]phenyl]-3,5,7-tris(acetylthiomethyl)adamantane (5). A solution of (4-ethynylphenyl)ethynylferrocene (**4**) (29.4 mg, 94.8 μmol) in THF (2.5 mL) was added to a mixture of 1-(4-iodophenyl)-3,5,7-tris(acetylthiomethyl)adamantane (**3**) (47.6 mg, 79.0 μmol), $Pd(PPh_3)_4$ (5.3 mg, 4.6 μmol), and CuI (3.2 mg, 17 μmol). Triethylamine (0.5 mL) was added, and the mixture was stirred at 60 °C for 20 h. After removal of solid materials by filtration through a layer of silica gel, the solution was washed with water and dried ($MgSO_4$). The solvent was evaporated, and the residue was purified by column chromatography (SiO_2, CH_2Cl_2–hexane 2:1) and gel permeation chromatography ($CHCl_3$) to give **5** as an orange solid (39.2 mg, 63%) with a melting point of 76.3–76.9 °C. ^{1}H NMR (300 MHz, $CDCl_3$) δ 7.47 (d, J = 7.3 Hz, 2H), 7.46 (s, 4H), 7.27 (d, J = 7.9 Hz, 2H), 4.50 (t, J = 1.8 Hz, 2H), 4.251 (t, J = 2.0 Hz, 2H), 4.246 (s, 5H), 2.85 (s, 6H), 2.36 (s, 9H) , 1.53 (s, 6H), 1.26 (s, 6H); ^{13}C NMR (75 MHz, $CDCl_3$) δ 195.2, 148.9, 131.5, 131.4, 131.2 125.0, 123.7, 122.4, 120.8, 90.9, 90.5, 89.0, 85.5, 71.4, 70.0, 68.9, 64.9, 45.3, 43.6, 41.0, 38.4, 35.8, 30.7; IR (ATR/ZnSe, cm^{-1}) 2917, 2219 ($\nu_{C\equiv C}$), 1682 ($\nu_{C=O}$), 1354, 1103; HRMS (MALDI-TOF) *m/z* calcd. for $C_{45}H_{44}FeO_3S_3$ $[M]^+$ 784.1802, found 784.1819.

1-[4-[4-(Ferrocenylethynyl)phenylethynyl]phenyl]-3,5,7-tris(mercaptomethyl)adamantane (2b). A MeOH solution (1 mL) of KOH (45 mg, 0.80 mmol) was added to a solution of tris(thioacetate) **5** (19.0 mg, 24.2 μmol) in a THF–MeOH mixed solvent (1:1, 6.0 mL). The reaction mixture was stirred at room temperature for 2 h and quenched by the addition of 10% HCl (1 mL). The product was extracted with $CHCl_3$, and the organic layer was washed with water and dried (Na_2SO_4). The solvent was evaporated, and the residue was purified by gel permeation chromatography ($CHCl_3$) to give **2b** as an orange solid (7.3 mg, 46%) with a melting point of 136.1–138.5 °C. ^{1}H NMR (500 MHz, $CDCl_3$) δ 7.44–7.50 (m, 6H), 7.35 (d, J = 8.5 Hz, 2H), 4.51 (t, J = 1.8 Hz, 2H), 4.26 (t, J = 1.8 Hz, 2H), 4.25 (s, 5H), 2.49 (d, J = 9.2 Hz, 6H), 1.60 (s, 6H), 1.34 (d, J = 12.1 Hz, 3H), 1.30 (d, J = 12.1 Hz, 3H), 1.21 (t,

J = 8.9 Hz, 3H); ^{13}C NMR (126 MHz, $CDCl_3$) δ 149.3, 131.5, 131.4, 131.2, 125.1, 123.7, 122.4, 120.8, 90.9, 90.5, 89.0, 85.5, 71.5, 70.0, 69.0, 64.9, 45.4, 43.1, 38.7, 37.5, 35.9; IR (NEAT/KRS-6, cm^{-1}) 2916, 2575 (ν_{S-H}), 2205 ($\nu_{C\equiv C}$), 1518 1354; HRMS (MALDI-TOF) *m*/*z* calcd. for $C_{39}H_{38}FeS_3$ $[M]^+$ 658.1480, found 658.1563.

References

[1] Kitagawa, T.; Idomoto, Y.; Matsubara, H.; Hobara, D.; Kakiuchi, T.; Okazaki, T.; Komatsu, K. *J. Org. Chem.* **2006**, *71*, 1362–1369.

[2] Kitagawa, T.; Matsubara, H.; Komatsu, K.; Hirai, K.; Okazaki, T.; Hase, T. *Langmuir* **2013**, *29*, 4275–4282.

[3] Hsung, R.P.; Chidsey, C.E.D.; Sita, L.R. *Organometallics* **1995**, *14*, 4808–4815.

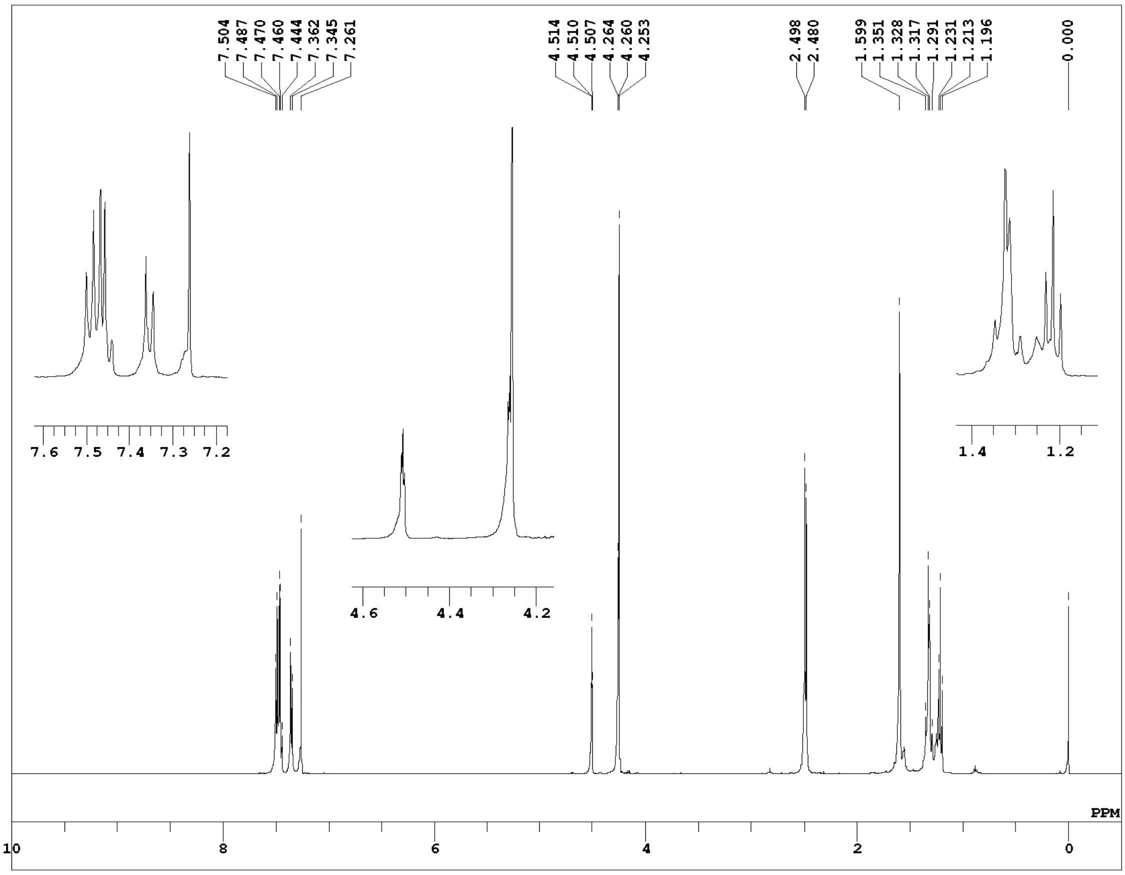

Figure S1. ^{1}H NMR spectrum of **5** (300 MHz, $CDCl_3$).

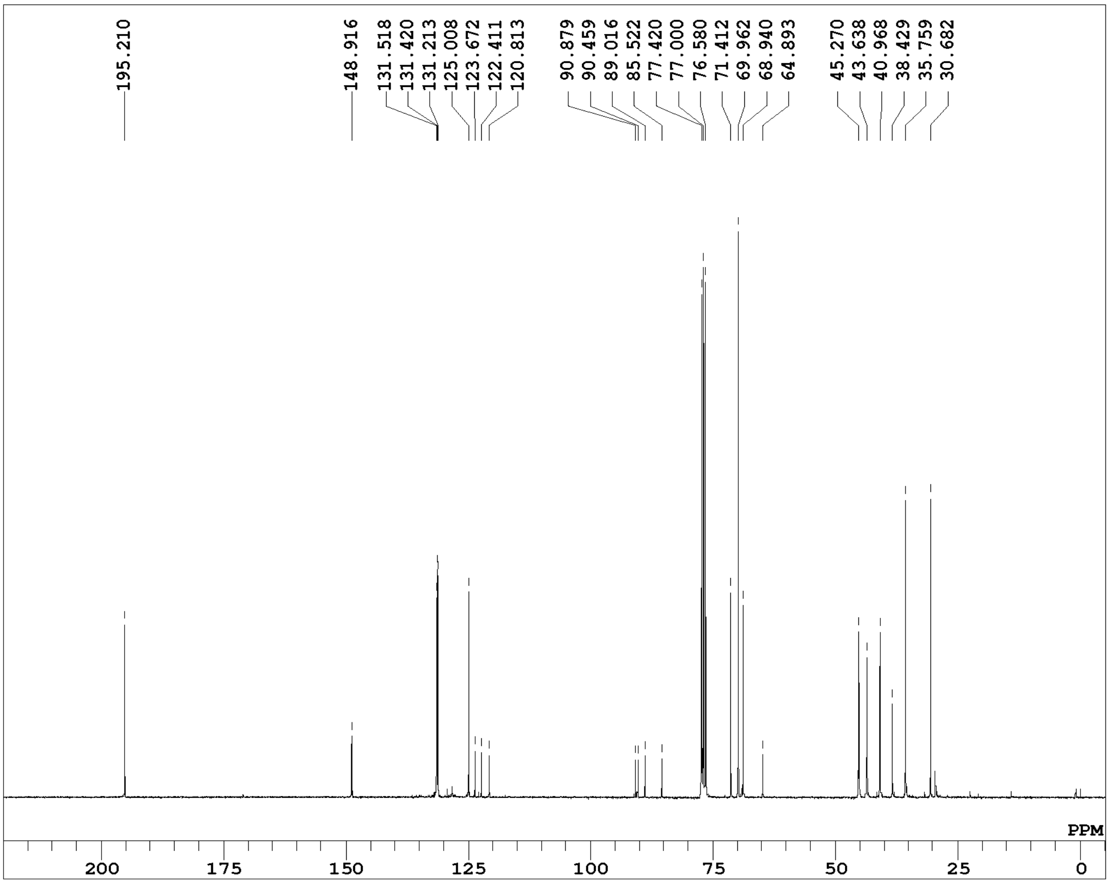

Figure S2. ^{13}C NMR spectrum of **5** (75 MHz, $CDCl_3$).

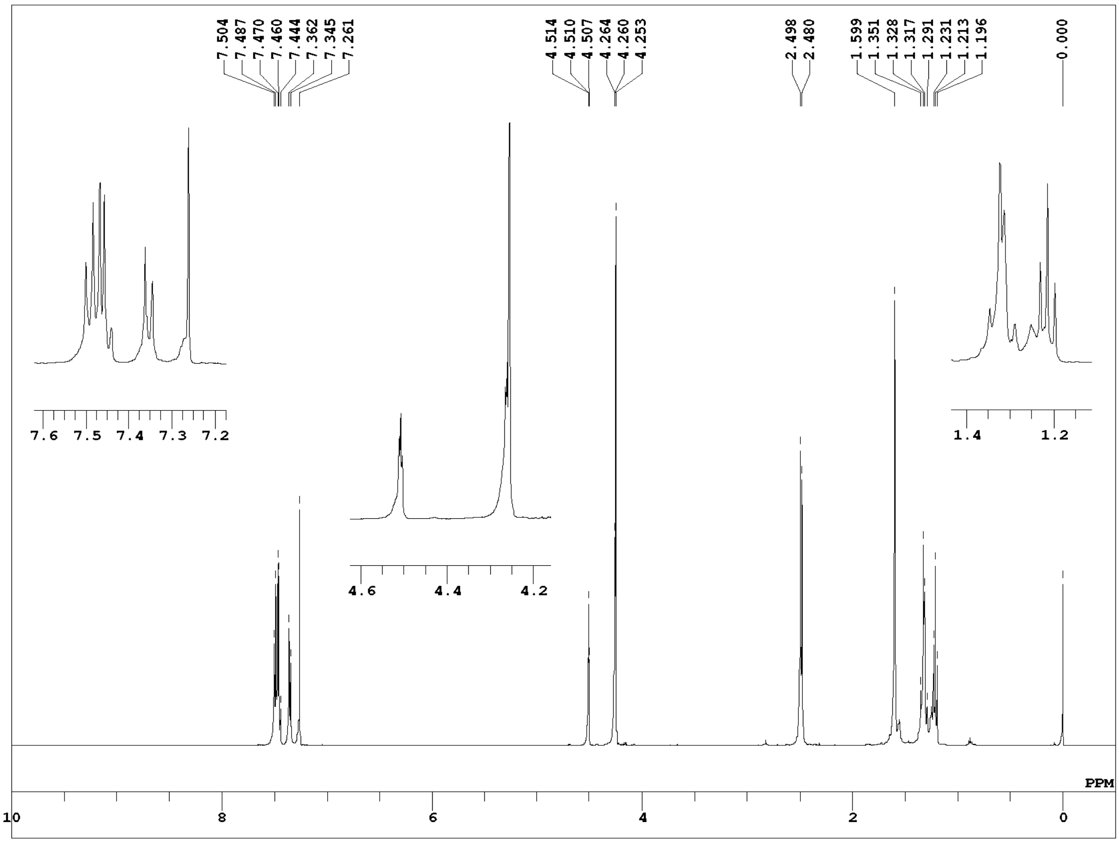

Figure S3. ^{1}H NMR spectrum of **2b** (500 MHz, $CDCl_3$).

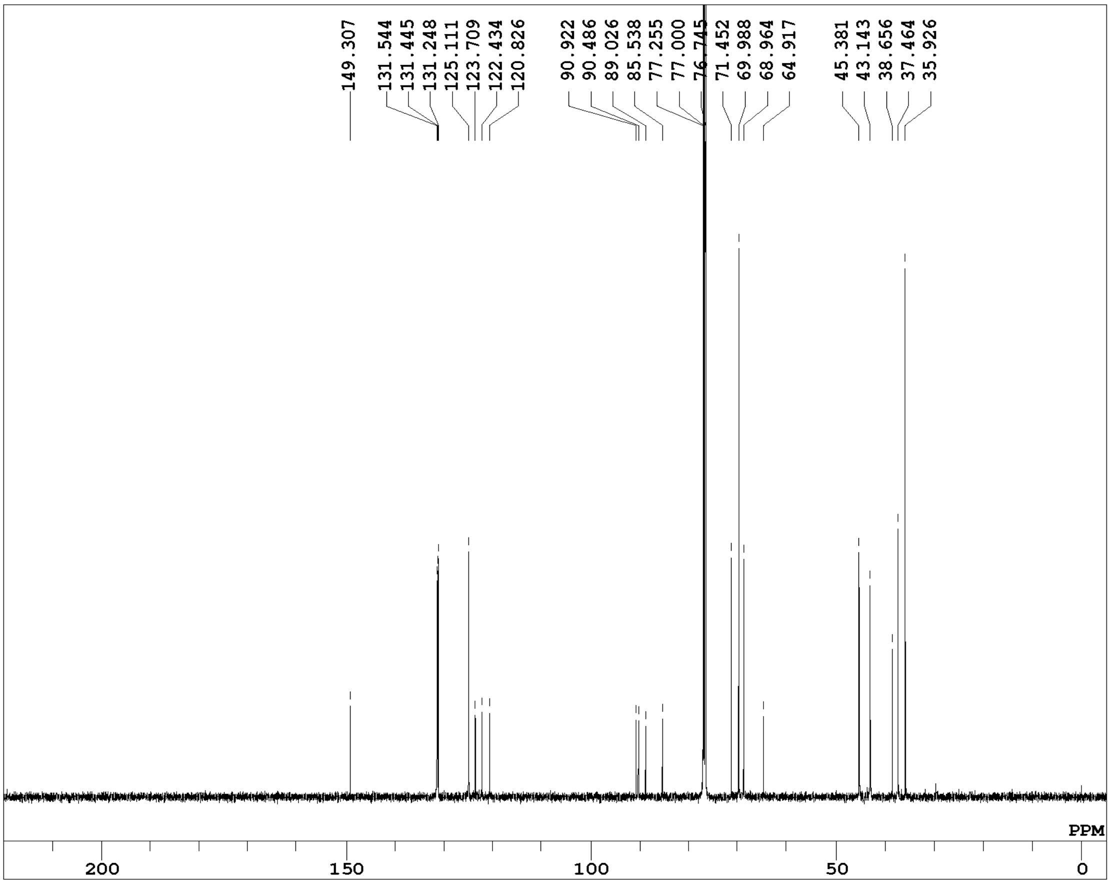

Figure S4. ^{13}C NMR spectrum of **2b** (126 MHz, $CDCl_3$).

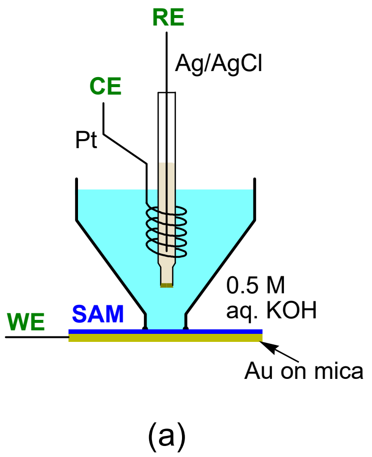

Figure S5. Cyclic voltammetry cells for reductive desorption (a) and oxidation of ferrocenyl group (b). The geometric area of the working electrode was 0.152 cm^2.

Table S1. Cartesian coordinates and energies for the DFT-optimized structure of **2b**.

Level of theory: B3LYP/3-21G(d)(C, H, and S), LANL2DZ(Fe)
HF = -2812.8637737 hartree
Number of imaginary frequencies: 0

Center Number	Atomic Number	Atomic Type	Coordinates (Angstroms) X	Y	Z
1	6	0	-7.727087	0.482740	1.407335
2	6	0	-8.292426	-0.888688	0.977203
3	6	0	-7.737075	-1.293106	-0.406046
4	6	0	-8.118211	-0.213330	-1.444368
5	6	0	-7.560029	1.167294	-1.030556
6	6	0	-8.116741	1.547539	0.358806
7	6	0	-6.189688	-1.376225	-0.315469
8	6	0	-6.178495	0.388632	1.474858
9	6	0	-6.013372	1.072666	-0.938553
10	6	0	-8.222444	0.869407	2.812340
11	16	0	-10.125257	0.971231	3.009999
12	6	0	-8.247737	-2.679645	-0.835198
13	16	0	-10.141720	-2.809312	-1.097961
14	6	0	-7.893047	2.246261	-2.075818
15	16	0	-9.759489	2.614181	-2.307521
16	6	0	-5.582524	-0.004337	0.099042
17	6	0	10.544577	-1.202518	1.270141
18	6	0	11.893394	-1.376267	0.825367
19	6	0	11.882540	-1.513566	-0.605725
20	6	0	10.526987	-1.425733	-1.055186
21	6	0	9.680603	-1.244508	0.107175
22	26	0	11.052471	0.355766	-0.056275
23	6	0	12.166945	1.911088	-0.959846
24	6	0	10.790916	1.992294	-1.368304
25	6	0	9.988712	2.177312	-0.190963
26	6	0	10.867283	2.208128	0.945199
27	6	0	12.214174	2.044413	0.469621
28	6	0	1.413809	-0.541058	0.105966
29	6	0	0.203104	-0.443698	0.109524
30	6	0	-1.908302	0.003864	1.292988
31	6	0	-3.297299	0.111419	1.294159
32	6	0	-4.049603	-0.109497	0.130257
33	6	0	-3.348117	-0.444337	-1.043202
34	6	0	-1.963577	-0.554003	-1.058597
35	6	0	-1.214339	-0.331211	0.115646
36	1	0	-8.011483	-1.648881	1.721454
37	1	0	-9.384803	-0.825375	0.943770
38	1	0	-9.209354	-0.171232	-1.523431
39	1	0	-7.710755	-0.493398	-2.427563
40	1	0	-9.206135	1.632593	0.292654
41	1	0	-7.709973	2.524806	0.659210
42	1	0	-5.785061	-1.679869	-1.288871
43	1	0	-5.897105	-2.134350	0.422739
44	1	0	-5.772136	1.359354	1.789300
45	1	0	-5.898776	-0.362040	2.226145
46	1	0	-5.596564	2.043541	-0.640199
47	1	0	-5.605870	0.819902	-1.925096
48	1	0	-7.935613	0.105705	3.540161

49	1	0	-7.807125	1.831017	3.124966
50	1	0	-10.290485	2.177496	2.373451
51	1	0	-7.962726	-3.445767	-0.109567
52	1	0	-7.840157	-2.949254	-1.813117
53	1	0	-10.488049	-2.848408	0.230975
54	1	0	-7.484927	1.984617	-3.055530
55	1	0	-7.483892	3.212285	-1.768425
56	1	0	-10.059325	1.486202	-3.032380
57	1	0	10.210810	-1.070978	2.286748
58	1	0	12.769690	-1.389099	1.453944
59	1	0	12.749409	-1.646546	-1.233443
60	1	0	10.178223	-1.488480	-2.073349
61	1	0	13.014152	1.763776	-1.611061
62	1	0	10.423330	1.917644	-2.379522
63	1	0	8.912250	2.243082	-0.161935
64	1	0	10.567650	2.323581	1.974846
65	1	0	13.103104	2.014510	1.079891
66	1	0	-1.348520	0.178427	2.203545
67	1	0	-3.793095	0.371146	2.219615
68	1	0	-3.893374	-0.623515	-1.962541
69	1	0	-1.445351	-0.813204	-1.973531
70	6	0	2.829705	-0.656054	0.104609
71	6	0	3.566547	-0.504949	1.299462
72	6	0	3.531331	-0.925326	-1.090601
73	6	0	4.949037	-0.618528	1.299189
74	1	0	3.036829	-0.299334	2.221152
75	6	0	4.913919	-1.038473	-1.091158
76	1	0	2.974589	-1.043556	-2.011717
77	6	0	5.651403	-0.887293	0.103761
78	1	0	5.505693	-0.502599	2.220735
79	1	0	5.443471	-1.246477	-2.012466
80	6	0	7.066775	-1.003247	0.103638
81	6	0	8.276793	-1.109933	0.104859
